# Efficacy and safety of antibody–drug conjugates in EGFR-mutated non-small cell lung cancer after EGFR-TKI resistance: a systematic review and meta-analysis

**DOI:** 10.3389/fonc.2026.1909040

**Published:** 2026-10-14

**Authors:** Yilin Chen, Shuaixuan Chen, Hang Zhang, Jiebo Xie, Binbin Song, Yufen Xu

**Affiliations:** 1 Zhejiang Chinese Medical University, Hangzhou, China; 2 First Hospital of Jiaxing, Jiaxing, Zhejiang, China

**Keywords:** antibody-drug conjugates, EGFR-mutated non-small cell lung cancer, EGFR-TKI resistance, meta-analysis, NSCLC, systematic review

## Abstract

**Background:**

Antibody–drug conjugates (ADCs) have emerged as potential treatment options for EGFR-mutated non-small cell lung cancer (NSCLC) after EGFR-tyrosine kinase inhibitor (TKI) resistance. We evaluated their efficacy and safety in this setting.

**Methods:**

PubMed, Embase,and the Cochrane Library were searched for prospective clinical studies. Overlapping reports were assessed before endpoint-specific synthesis. Comparable proportional outcomes from non-randomized studies were pooled using random-effects models, whereas randomized controlled trials (RCTs) were summarized separately.

**Results:**

Eight eligible clinical study reports were included. Four non-randomized reports contributed five independent underlying trial datasets; however, the ORR and DCR meta-analysis each included four non-overlapping analytical estimates because one pooled Dato-DXd estimate represented two underlying trial datasets that were not entered separately. The pooled ORR was 39.2%(95% CI,31.1%–48.0%),and the pooled DCR was 78.9%(95% CI,72.3%–84.2%).Three comparable non-overlapping datasets contributed to the grade ≥3 TEAE analysis, yielding a pooled incidence of 65.2%(95% CI,50.2%–77.7%),whereas four analytical datasets contributed to the ILD analysis, with a pooled reported incidence of 6.0%(95% CI,4.1%–8.5%).Two RCTs provided comparative evidence supporting sac-TMT, particularly for ORR and PFS.

**Conclusions:**

ADCs showed antitumor activity after EGFR-TKI resistance, but efficacy and toxicity varied across platforms. Additional randomized trials and biomarker-informed studies are needed to define the optimal use of individual ADCs.

**Systematic review registration:**

https://www.crd.york.ac.uk/PROSPERO/view/CRD420261288638, identifier CRD420261288638.

## 1 Introduction

### 1.1 Current treatment landscape and challenges of drug resistance in EGFR-mutated NSCLC

The identification of activating epidermal growth factor receptor (EGFR) mutations has substantially changed the treatment paradigm for EGFR-mutated non-small cell lung cancer (NSCLC) (1, 2). Third-generation EGFR tyrosine kinase inhibitors (EGFR-TKIs), particularly osimertinib, have become standard first-line therapy for advanced EGFR-mutated NSCLC and have demonstrated clear benefits in progression-free survival (PFS) and overall survival (OS) (3).

Despite these advances, acquired resistance eventually develops in nearly all patients with advanced EGFR-mutated NSCLC who initially benefit from EGFR-TKI therapy (4, 5).This process is driven by clonal evolution, phenotypic remodeling, and adaptive tumor reprogramming under sustained drug-selective pressure (6). Resistance mechanisms are highly heterogeneous and include secondary EGFR mutations as well as EGFR-independent escape pathways, such as MET amplification, HER2/HER3 bypass activation, downstream signaling reprogramming, and small-cell transformation (7). These mechanisms frequently coexist and evolve dynamically, which limits the durability of single-target inhibition after resistance has emerged and creates major challenges for subsequent treatment.

After EGFR-TKI failure, platinum-based doublet chemotherapy remains one of the most commonly used subsequent-line treatments in clinical practice (8, 9). However, response rates are modest, disease control is often short-lived, and cumulative toxicity from multiple lines of therapy can further limit clinical benefit (2). The role of immunotherapy in EGFR-mutated NSCLC also remains uncertain (10). Fang et al. (11) reported that, among patients whose disease progressed during third-generation EGFR-TKI therapy, ivonescimab plus chemotherapy improved PFS compared with control therapy (median PFS, 7.1 vs. 4.8 months; 95% CI, 0.35-0.66), suggesting that PD-1/VEGF bispecific antibodies may represent a promising strategy for EGFR-TKI-resistant disease. However, long-term survival benefit, optimal sequencing, and appropriate patient selection require further clarification. Therefore, substantial unmet clinical needs remain for later-line treatment, and more effective, durable, and biologically rational strategies are needed after EGFR-TKI resistance.

### 1.2 Biological basis and therapeutic potential of antibody-drug conjugates

Antibody-drug conjugates (ADCs) (12, 13) have emerged as a novel therapeutic approach for patients with EGFR-TKI-resistant disease. Unlike EGFR-TKIs, which primarily inhibit the EGFR kinase domain, ADCs use an antibody to recognize tumor-associated surface antigens and deliver highly potent cytotoxic payloads into tumor cells, thereby enabling selective tumor cell killing (12). This mechanism may allow ADC activity to remain partly independent of EGFR kinase-domain mutational status, providing a potential advantage in the resistance setting (14).

Structurally, ADCs consist of three interdependent components: a monoclonal antibody that determines target recognition, a linker that controls systemic stability and payload release, and a highly potent cytotoxic payload. The clinical performance of an ADC is influenced not only by target expression but also by target density and internalization kinetics, antibody specificity, linker chemistry, payload potency and membrane permeability, drug-to-antibody ratio, and the capacity to generate a bystander effect (15, 16). Consequently, optimization of ADC design requires coordinated engineering of all three components rather than target selection alone. Recent developments in CD20-directed ADCs further illustrate this principle: slow target internalization can limit conventional ADC activity, whereas strategies such as cleavable linkers, membrane-permeable payloads, site-specific conjugation, and alternative conjugate platforms may help overcome target-specific delivery barriers (17).

Several ADCs, including patritumab deruxtecan, datopotamab deruxtecan, sacituzumab tirumotecan, and telisotuzumab vedotin, have shown encouraging clinical activity in EGFR-mutated NSCLC after EGFR-TKI resistance (14, 18). These findings suggest that ADCs may become an important addition to conventional later-line treatment. Some agents have advanced into randomized clinical trials and have received regulatory approval, indicating that ADCs are moving from exploratory approaches toward clinically translatable therapeutic platforms (19).

These design considerations underscore that ADCs should not be regarded as a homogeneous therapeutic class (20). In EGFR-TKI-resistant NSCLC, this heterogeneity extends beyond ADC architecture to the biology of the target itself. HER3 expression has been reported to increase in paired tumors after acquired EGFR-TKI resistance (21), whereas MET amplification or pathway activation is a recognized bypass-resistance mechanism (22). Direct paired clinical evidence for treatment-induced TROP2 upregulation remains limited, although TROP2 may be retained in drug-tolerant persister states (23). In addition, differences in bystander effects, payload release kinetics, and toxicity profiles further complicate clinical selection across ADC platforms.

Systematically integrated evidence remains limited regarding the clinical value, efficacy heterogeneity, and safety profiles of different ADCs in EGFR-TKI-resistant NSCLC. The clinical positioning of ADCs also remains unclear across study designs, targets, and treatment contexts. Therefore, an evidence-based evaluation of ADC efficacy and safety is needed to inform subsequent-line treatment decisions.

### 1.3 Objectives of the study

This study was conducted to systematically evaluate the efficacy and safety of adcs in patients with egfr-mutated nsclc after egfr-tki resistance and to synthesize the strength of evidence across different study designs. The outcomes of interest included orr, dcr, pfs, os, and clinically relevant safety outcomes, particularly high-grade adverse events and ild.

Because the available evidence was derived from heterogeneous study designs, single-arm studies and randomized controlled trials were interpreted separately according to the hierarchy of evidence. Single-arm studies were used to estimate the absolute efficacy and safety profiles of adcs, whereas randomized controlled trials were used to evaluate the comparative benefit of sacituzumab tirumotecan versus standard therapy. By synthesizing current clinical evidence, this study aimed to provide evidence-based support for adc selection in later-line treatment of egfr-mutated nsclc and to inform future individualized treatment strategies.

## 2 Materials and methods

### 2.1 Study design and reporting guidelines

This study was conducted in accordance with the 2020 Preferred Reporting Items for Systematic Reviews and Meta-Analyses (PRISMA) statement (24) and was prospectively registered in PROSPERO (CRD420261288638) to ensure transparency, standardization, and reproducibility. The efficacy and safety of antibody-drug conjugates (ADCs) in patients with EGFR-mutated non-small cell lung cancer (NSCLC) after EGFR tyrosine kinase inhibitor (EGFR-TKI) failure were systematically evaluated, and evidence from single-arm studies and randomized controlled trials was assessed separately.

### 2.2 Literature search strategy

PubMed, Embase, and the Cochrane Library were systematically searched from database inception to January 19, 2026.The search strategy was intentionally broad and sensitivity-oriented, combining database-specific controlled-vocabulary and free-text terms related to NSCLC, advanced or recurrent disease, ADCs, and clinical trials. EGFR mutation- and EGFR-TKI-related terms were not required in the electronic search strings to reduce the risk of missing potentially eligible studies in which EGFR-mutated populations were reported only as subgroups or pooled subsets. EGFR mutation status and prior EGFR-TKI exposure were subsequently assessed during study screening according to the prespecified eligibility criteria. Reference lists of relevant studies were also screened to identify additional potentially eligible reports.The complete database-specific search strategies are provided in **Supplementary Appendix S1**. The exported records from the original Cochrane Library search are additionally provided as **Supplementary Data Sheet S1**.

### 2.3 Eligibility criteria

Inclusion and exclusion criteria were prespecified according to the PICOS framework (Population, Intervention, Comparison, Outcomes, and Study Design). Eligible participants were adults with advanced (stage III-IV) EGFR-mutated NSCLC who had experienced disease progression after EGFR-TKI therapy. Eligible interventions included ADC monotherapy or ADC-based regimens. Comparators, when applicable, included standard chemotherapy or other conventional subsequent-line therapies. Efficacy outcomes included objective response rate (ORR), progression-free survival (PFS), overall survival (OS), and disease control rate (DCR).Safety outcomes included treatment-emergent adverse events (TEAEs), treatment-related adverse events (TRAEs), grade ≥3 safety events, and interstitial lung disease (ILD), as defined in the original studies.

Studies were eligible if they reported extractable data for at least one prespecified efficacy or safety outcome in the eligible population. Reports that did not provide extractable data for any prespecified outcome were excluded. The absence of PFS or OS data alone was not considered an exclusion criterion when other eligible outcomes were available.

Eligible studies were limited to prospective clinical trials published in English, including randomized controlled trials and single-arm clinical trials. Studies were excluded if they involved non-NSCLC populations, non-ADC interventions, EGFR mutation types outside the prespecified scope, pediatric populations, unavailable full text, or non-extractable outcomes. Reviews, case reports, observational studies, editorials, expert opinions, conference abstracts, expanded-access programs, and non-English publications were also excluded. Phase I dose-escalation or expansion studies were included when they demonstrated clear efficacy signals and reported systematic safety outcomes, because they were considered to provide single-arm evidence comparable to phase II-level data.Published reports from the same clinical trial were considered separately during study identification but were assessed for overlapping populations before quantitative synthesis.

### 2.4 Study selection process

Study selection was performed according to the PRISMA framework. All search records were imported into EndNote 20 for deduplication. Two reviewers independently screened titles and abstracts, followed by full-text review for eligibility. Outcomes from eligible studies were extracted, categorized, and organized according to study design. Disagreements were resolved through discussion, and a third reviewer was consulted when consensus could not be reached.

### 2.5 Data extraction

Data were independently extracted by two reviewers using a standardized data extraction form. The following information was collected:

1. Study characteristics: first author, publication year, study design, and trial phase;
2. Patient characteristics: sample size, EGFR mutation subtype, and prior treatment history;
3. Treatment characteristics: ADC agent, target, dose, and administration schedule;
4. Efficacy outcomes: ORR, DCR, PFS, and OS;
5. Safety outcomes: treatment-emergent adverse events (TEAEs), treatment-related adverse events (TRAEs), grade ≥3 safety events, and ILD

When reported data were incomplete, **Supplementary Materials** were reviewed or study authors were contacted to obtain relevant information.

### 2.6 Quality assessment

Risk of bias in randomized controlled trials was assessed using the revised Cochrane Risk of Bias Tool for randomized trials (RoB 2.0) (25). The assessed domains included the randomization process, deviations from intended interventions, missing outcome data, outcome measurement, and selective reporting. Each domain was judged as “low risk,” “some concerns,” or “high risk,” and an overall risk-of-bias judgment was then assigned.

Single-arm and nonrandomized studies were evaluated using the Methodological Index for Non-Randomized Studies (MINORS) (26). The eight MINORS items included clarity of the study aim, consecutive patient inclusion, prospective data collection, appropriateness of endpoints, unbiased endpoint assessment, adequacy of follow-up, control of loss to follow-up, and justification of sample size. Each item was scored from 0 to 2, with 0 indicating no reporting, 1 indicating inadequate reporting, and 2 indicating adequate reporting.

The overall certainty of the evidence was assessed using the Grading of Recommendations Assessment (27), Development and Evaluation (GRADE) approach. Evidence certainty was evaluated across five domains: risk of bias, inconsistency, imprecision, indirectness, and publication bias (28). The certainty of evidence was classified as high, moderate, low, or very low (29).

### 2.7 Outcomes of interest

The primary efficacy outcomes were objective response rate (ORR), disease control rate (DCR), and progression-free survival (PFS). Secondary outcomes included overall survival (OS), duration of response (DOR), and clinically relevant safety outcomes, including treatment-emergent adverse events (TEAEs), treatment-related adverse events (TRAEs), grade ≥3 safety events, and interstitial lung disease (ILD), according to the definitions used in the original studies.

### 2.8 Statistical analysis

Given the expected clinical and methodological heterogeneity across study designs, ADC targets, payload structures, treatment settings, and prior treatment exposure, a stratified analytical approach was used. Non-randomized datasets were used to estimate absolute efficacy and safety event rates, whereas randomized controlled trials (RCTs) were analyzed separately to provide comparative evidence. Because the included ADC platforms differed in molecular target, antibody structure, linker system, payload, and toxicity profile, pooled estimates were interpreted as class-level summary estimates rather than as direct comparisons among individual ADC agents. Random-effects models were used for all quantitative syntheses.

For non-randomized evidence, quantitative synthesis was performed at the dataset level rather than the publication level. Earlier reports, updated analysis, subgroup analysis, and pooled reports originating from the same or related clinical trial populations were assessed for potential overlap before meta-analysis. Dataset selection was performed separately for each endpoint according to the completeness of outcome reporting, size of the evaluable population, maturity of follow-up, and relevance to the outcome of interest. After overlap assessment, five independent underlying non-randomized trial datasets were identified across four clinical reports. Endpoint-specific meta- analysis were subsequently performed using non-overlapping analytical estimates according to outcome availability and comparability. When a pooled analytical estimate was used for a given endpoint, its contributing underlying trial datasets were not entered separately into the same meta-analysis.

For proportional outcomes, including objective response rate (ORR), disease control rate (DCR), grade ≥3 safety outcomes, and interstitial lung disease (ILD), proportions were logit-transformed and pooled using random-effects models when outcome definitions were considered clinically comparable. Between-dataset variance was estimated using the restricted maximum likelihood method. Safety outcomes were analyzed according to the definitions used in the original studies. In particular, grade ≥3 treatment-emergent adverse events (TEAEs) and treatment-related adverse events (TRAEs) were evaluated separately, and datasets reporting clinically different safety endpoints were not combined within the same meta-analysis.

For comparative time-to-event outcomes from RCTs, trial-reported hazard ratios (HRs) with 95% confidence intervals (CIs) were extracted and summarized by study. Because non-randomized single-arm studies lacked an internal comparator, their progression-free survival (PFS), overall survival (OS), and duration of response (DOR) outcomes were summarized narratively using the reported Kaplan–Meier medians and 95% CIs rather than pooled as comparative HRs. When survival outcomes were immature or incompletely reported, the available findings were summarized narratively.

Between-dataset heterogeneity was assessed using Cochran’s Q test, the I² statistic, and the estimated between-dataset variance, and was interpreted jointly with clinical and methodological differences among datasets. Exploratory subgroup analysis according to ADC target were performed when sufficient data were available. Because of the limited number of analytical estimates within individual subgroups, subgroup findings were considered hypothesis-generating. Sensitivity analysis were performed, where feasible, by sequentially excluding individual datasets. Additional endpoint-specific sensitivity analysis were conducted when differences in outcome definitions or assessment frameworks could affect comparability.

Formal assessment of publication bias using funnel plots or Egger’s regression test was not performed because each pooled analysis included only three or four analytical datasets, making statistical assessment of funnel plot asymmetry unreliable.

RCT findings were synthesized narratively by study and endpoint because the control regimens, treatment settings, study phases, and endpoint maturity differed across trials. Trial-reported comparative measures were summarized when available, with HRs used for time-to-event outcomes and relative or absolute between-group effects used for dichotomous outcomes. Cross-trial quantitative pooling was not performed. A network meta-analysis was also not conducted because only two eligible RCTs were available, both evaluating sac-TMT but against different control regimens and in different treatment settings. Each direct comparison was informed by a single trial, no closed evidence loop was available, and clinical differences between trials raised concerns regarding the transitivity assumption. The remaining ADC evidence was derived from non-randomized single-arm datasets without common randomized comparators and was therefore unsuitable for inclusion in a conventional network meta-analysis.

## 3 Results

### 3.1 Literature search results and quality assessment

#### 3.1.1 Literature search results

The study selection process followed the PRISMA 2020 guidelines (**Figure 1**). A total of 635 records were identified through searches of PubMed (n = 105), the Cochrane Library (n = 197), and Embase (n = 333). After deduplication using EndNote 20 and manual review, 138 duplicate records were removed. Titles and abstracts of the remaining 497 records were screened, and 462 records were excluded because they did not meet the inclusion criteria. The main reasons for exclusion were reviews or systematic reviews (n = 88), studies of nonadvanced NSCLC (n = 36), ineligible clinical trial phase (n = 74), ineligible population (non-EGFR-mutated or non-NSCLC; n = 196), and ineligible publication type, including editorials, case reports, and conference abstracts (n = 68). The remaining 35 full-text records were assessed for eligibility. Of these, 27 were excluded because they provided no extractable data for any prespecified outcome in the eligible population (n = 17), focused on background or mechanistic topics (n = 2), or evaluated an ineligible non-ADC intervention (n = 8).Finally, eight eligible clinical study reports representing independent clinical evidence sources were included in the systematic review. Several reports represented updated analysis, subgroup analysis, or pooled datasets from overlapping trial populations. These relationships were carefully evaluated before quantitative synthesis, and endpoint-specific non-overlapping datasets were selected for meta-analysis.

**Figure 1:**
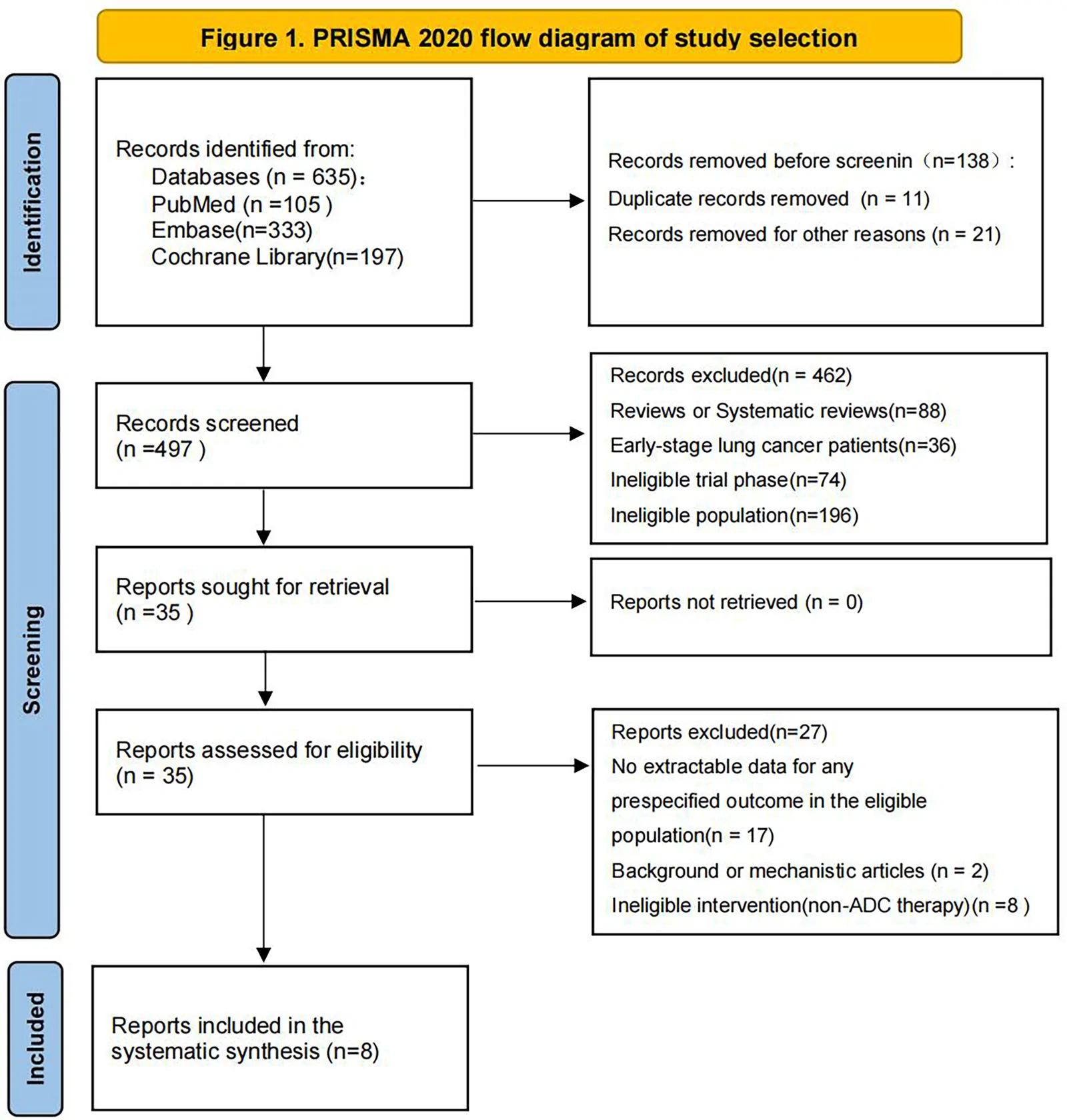
PRISMA 2020 flow diagram illustrating the study selection process. A total of 635 records were identified from PubMed (105), Embase (333), and the Cochrane Library (197). After removing 138 records before screening, 497 records underwent title and abstract screening, of which 462 were excluded. Thirty-five full-text reports were assessed for eligibility. Twenty-seven reports were excluded because of unavailable extractable outcomes (n = 17), background or mechanistic content (n = 2), or ineligible non-ADC interventions (n = 8). Ultimately, eight eligible clinical study reports were included in the systematic review.

#### 3.1.2 Study quality and risk of bias

The two included randomized controlled trials were assessed using RoB 2.0. Overall, both trials had a relatively clear randomization process; however, because of the open-label design and incomplete reporting of allocation concealment, the overall risk of bias was judged as having some concerns. The non-randomized clinical reports were evaluated using MINORS, with total scores ranging from 14 to 16, indicating generally good methodological quality. All studies performed well in clarity of study aims, consecutive patient inclusion, prospective data collection, and endpoint definition. The main limitations were the absence of sample size justification and insufficient reporting of loss to follow-up in some studies (**Figure 2**; **Table 1**).

**Figure 2:**
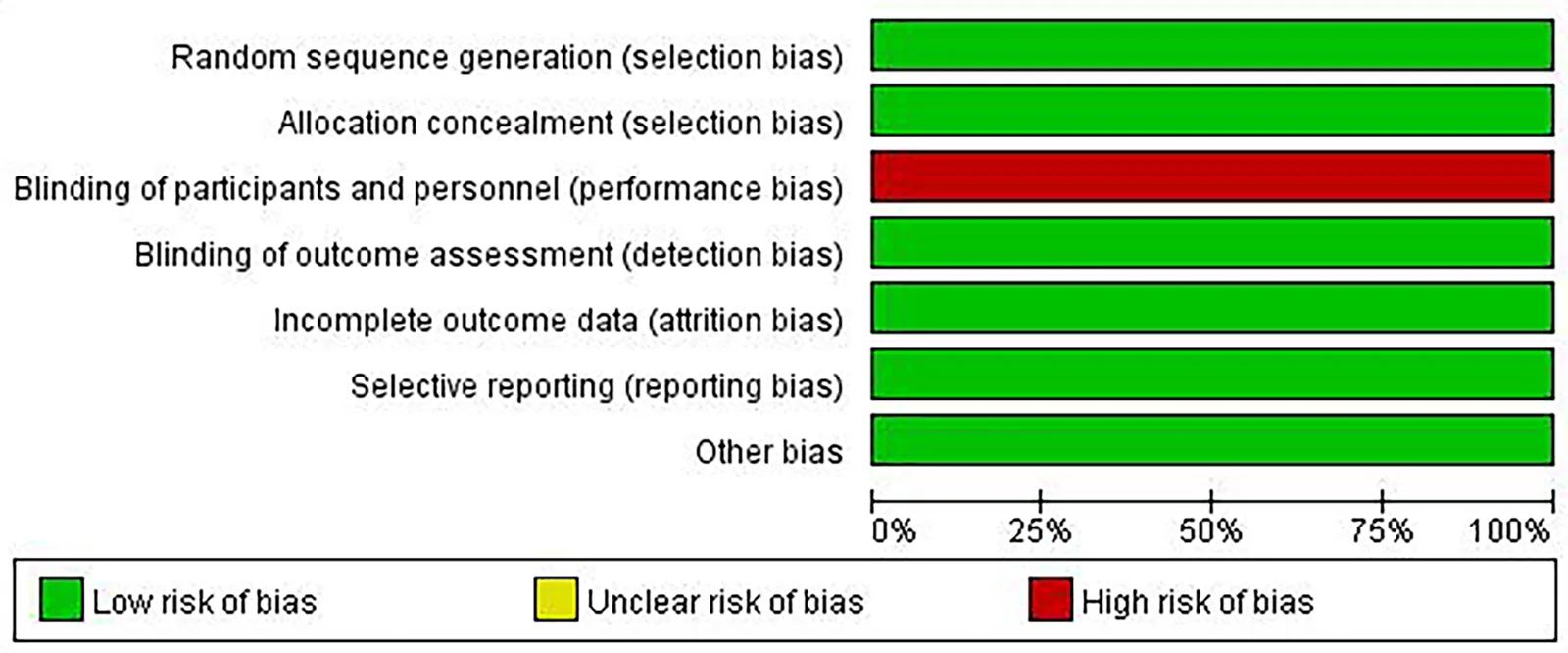
Risk-of-bias assessment of randomized controlled trials using the RoB 2.0 tool. The risk of bias in the included randomized controlled trials was assessed using the revised Cochrane Risk of Bias Tool for randomized trials (RoB 2.0). Each domain was judged as low risk, some concerns, or high risk. Green indicates low risk of bias, yellow indicates some concerns, and red indicates high risk of bias.

**Table 1:** Methodological quality assessment of single-arm and nonrandomized studies using the MINORS tool.

Author & year	1. Aim	2. Consecutive inclusion	3. Prospective	4. Endpoints	5. Unbiased	6. Follow-up	7. Loss < 5%	8. Sample size calculation	Total score
Jacob Sands 2025 (18)	2	2	2	2	2	2	2	1	15
Pasi A Jänne 2022 (14)	2	2	2	2	2	2	2	1	15
Helena A. Yu et al., 2023 (30)	2	2	2	2	2	2	2	2	16
Myung-Ju Ahn 2025 (31)	2	2	2	2	2	2	2	1	15
H Horinouchi 2025 (33)	2	2	2	2	1	2	2	1	14
H A Yu 2024 (32)	2	2	2	2	2	2	2	1	15

0: Not reported; 1: Reported but inadequate; 2: Reported and adequate.

MINORS, Methodological Index for Non-Randomized Studies. The eight MINORS items included a clearly stated aim, consecutive patient inclusion, prospective data collection, appropriate endpoints, unbiased endpoint assessment, adequate follow-up, loss to follow-up less than 5%, and prospective sample size calculation. Each item was scored as 0, not reported; 1, reported but inadequate; or 2, reported and adequate. The maximum total score for noncomparative studies was 16, with higher scores indicating better methodological quality.

#### 3.1.3 Certainty of evidence assessment

The certainty of evidence was assessed using the GRADE framework on an outcome-specific basis. The certainty of evidence for pooled non-randomized outcomes, including ORR, DCR, grade ≥3 TEAEs, and ILD, was rated as very low because of outcome-specific concerns related to risk of bias, inconsistency, indirectness, and/or imprecision. In particular, heterogeneity across ADC platforms and clinical settings contributed to concerns regarding inconsistency and indirectness, whereas the limited number of available datasets reduced the precision of some estimates. By contrast, randomized comparative evidence was rated as moderate, primarily because of some concerns regarding risk of bias related to the open-label study design and other trial-level methodological limitations. Detailed outcome-specific judgments and reasons for downgrading are provided in **Table 2**.

**Table 2:** GRADE assessment of the certainty of evidence for ADCs in EGFR-mutated NSCLC after EGFR-TKI resistance.

Outcome	Evidence source	No. of datasets/studies	Effect estimate	Risk of bias	Inconsistency	Indirectness	Imprecision	Certainty of evidence
Overall response rate (ORR)	Non-randomized datasets	4 datasets from 4 non-randomized reports	Pooled ORR: 39.2% (95% CI, 31.1%–48.0%)	Serious	Serious	Serious	No serious	Very low
Disease control rate (DCR)	Non-randomized datasets	4 datasets from 4 non-randomized reports	Pooled DCR: 78.9% (95% CI, 72.3%–84.2%)	Serious	Serious	Serious	No serious	Very low
Grade ≥3 treatment-emergent adverse events (TEAEs)	Non-randomized datasets	3 non-overlapping analytical datasets	Pooled incidence: 65.2% (95% CI, 50.2%–77.7%)	Serious	Serious	Serious	serious	Very low
Interstitial lung disease (ILD)	Non-randomized datasets	4 analytical datasets	Pooled incidence: 6.0% (95% CI, 4.1%–8.5%)	Serious	No serious	Serious	Serious	Very low
Comparative efficacy of sac-TMT versus control therapy	Randomized controlled trials	2 RCTs	HR/RR estimates according to reported endpoints	Some concerns	No serious	No serious	No serious	Moderate

Certainty of evidence was assessed according to the GRADE framework on an outcome-specific basis. Non-randomized evidence was initially rated as low certainty and was further downgraded when serious concerns were identified regarding risk of bias, inconsistency, indirectness, or imprecision. Randomized controlled trial evidence was initially rated as high certainty and was downgraded to moderate because of some concerns related to open-label study design and other trial-level methodological limitations. Formal statistical assessment of publication bias was not performed because each pooled analysis included only three to four analytical datasets; therefore, publication bias could not be reliably assessed and cannot be excluded.

### 3.2 Baseline characteristics of included studies

Eight eligible clinical study reports were included and are summarized in **Table 3**. Because several publications represented earlier reports, updated analysis, nested subgroups, or pooled analysis of related trial populations, baseline characteristics were presented according to the analytical unit reported in each source, including study reports, trial datasets, cohorts, subgroups, pooled populations, and treatment arms. Accordingly, sample sizes were not summed to estimate the total number of unique participants.

**Table 3:** Baseline characteristics and overlap-handling framework of included clinical study reports and patient populations.

Study report	Underlying trial/dataset	Relationship with other reports	Included in quantitative synthesis	Study design/phase	Trial ID (NCT)	ADC drug/dose	Target	Sample size, n	Median age, years (range)	Female (n, %)	Brain metastases (n, %)	Prior osimertinib use (n, %)	Prior platinum chemotherapy (n, %)	EGFR mutation subtype, n (%)	Remarks
TROPION-Lung05 (Jacob Sands 2025) (18)	Overall study cohort	Original TROPION-Lung05 report; overlaps with the EGFRm TROPION-Lung05 subset (n = 78) included in the later Ahn 2025 pooled report	No for overlapping endpoints when the pooled analytical estimate was used	Single-arm, phase II	NCT04484142	Datopotamab Deruxtecan (6.0 mg/kg Q3W)	TROP2	137	61 (29–79)	83 (60.6%)	70 (51.1%)	61 (44.5%)	137 (100%)	Ex19del: 41 (29.9%) L858R: 25 (18.2%) Others: 36 (26.3%)	Overall TROPION-Lung05 cohort with actionable genomic alterations
U31402-A-U102 (Pasi A. Jänne 2022) (14)	Early dose-escalation cohort	Earlier report from the same underlying trial as the Yu HA 2024 updated analysis (NCT03260491)	No; replaced by the updated dataset for overlapping endpoints	Dose-escalation/expansion, phase I	NCT03260491	Patritumab Deruxtecan (5.6 mg/kg Q3W)	HER3	57	65 (40–80)	36 (63.2%)	9 (15.8%)	49 (86.0%)	52 (91.2%)	Ex19del: 33 (57.9%) L858R: 20 (35.1%) Others: 8 (14.0%)	Early report
HERTHENA-Lung01 (Helena A. Yu 2023) (30)	Overall cohort	Independent report/dataset; the osimertinib subgroup below is nested within this overall cohort	Yes; endpoint-specific	Single-arm, phase II	NCT04619004	Patritumab Deruxtecan (5.6 mg/kg Q3W)	HER3	225	64 (37–82)	132 (58.7%)	72 (32.0%)	209 (92.9%)	225 (100%)	Ex19del: 142 (63.1%) L858R: 81 (36.0%) Others: 1 (0.4%)	Overall cohort
Pooled Analysis (Myung-Ju Ahn 2025) (31)	Overall pooled EGFRm population	Pooled analytical population comprising two independent underlying trial datasets: TROPION-Lung05 EGFRm (n = 78) and TROPION-Lung01 EGFRm Dato-DXd (n = 39)	Yes; used as one pooled analytical estimate for selected endpoints	Pooled analysis, phase II/III	NCT04484142 & NCT04656652	Datopotamab Deruxtecan (6 mg/kg Q3W)	TROP2	117	63 (36–81)	73 (62%)	36 (31%)	96 (82%)	116 (99%)	Ex19del: 60 (51%) L858R: 37 (32%) Multiple EGFR mutations permitted*	Combined analytical population
OptiTROP-Lung04 (W. Fang 2025) (34)	Sac-TMT arm	Treatment arm of OptiTROP-Lung04; paired with the control arm below	Analyzed separately as comparative RCT evidence; not pooled with non-randomized proportions	RCT, phase III	NCT05870319	Sacituzumab Tirumotecan (5 mg/kg, d1,15, Q28d)	TROP2	188	60 (31–75)	122 (64.9%)	33 (17.6%)	178 (94.7%)	Not reported	Ex19del: 106 (56.4%) L858R: 84 (44.7%) Others: 8 (4.3%)	Treatment arm
OptiTROP-Lung03 (Fang W 2025) (34)	Sac-TMT arm	Treatment arm of OptiTROP-Lung03; paired with the control arm below	Analyzed separately as comparative RCT evidence; not pooled with non-randomized proportions	RCT, phase II	NCT05613262	Sacituzumab Tirumotecan (5 mg/kg, d1,15, Q28d)	TROP2	91	57 (37–75)	53 (58.2%)	18 (19.8%)	84 (92.3%)	91 (100%)	Ex19del: 43 (47.3%) L858R: 48 (52.7%) Others: 0	Treatment arm
U31402-A-U102 (Yu HA 2024) (32)	Overall updated cohort	Updated analysis of the same underlying trial as the Jänne 2022 early report	Yes; endpoint-specific	Dose-escalation/expansion, phase I	NCT03260491	Patritumab Deruxtecan (5.6 mg/kg Q3W)	HER3	97	64 (40–80)	66 (68.0%)	26 (26.8%)	77 (79.4%)	97 (100%)	Ex19del: 64 (66.0%) L858R: 29 (29.9%) Others: 4 (4.1%)	Updated report
Teliso-V Study (H. Horinouchi 2025) (33)	Arm E cohort	Independent cohort	Yes; endpoint-specific	Single-arm, phase I/Ib	NCT02099058	Telisotuzumab Vedotin (1.6 mg/kg or 1.9 mg/kg Q2W)	c-MET	38	60 (40–79)	25 (65.8%)	7 (18.4%)	38 (100%)	18 (47.4%)	Ex19del: 19 (50.0%) L858R: 18 (47.4%) Others: 1 (2.6%)	c-MET overexpression cohort

Values are presented as n (%) unless otherwise indicated. ADC, antibody-drug conjugate; EGFR, epidermal growth factor receptor; EGFRm, EGFR-mutated; NCT, ClinicalTrials.gov identifier; Q3W, every 3 weeks; Q2W, every 2 weeks; Q28d, every 28 days; RCT, randomized controlled trial. Sample sizes are presented at the study-report, underlying trial/dataset, subgroup, pooled-population, or treatment-arm level and were not summed to estimate the number of unique participants because some reports represent updated analysis, nested subgroups, or pooled analyses from overlapping trial populations.

The Ahn 2025 pooled EGFRm population (N = 117) comprises two independent underlying trial datasets: TROPION-Lung05 (n = 78) and TROPION-Lung01 Dato-DXd (n = 39); the pooled population is therefore an analytical combination of these two datasets and should not be counted as a third independent dataset. For quantitative synthesis, overlap was assessed at both the dataset and endpoint levels. When a pooled analytical estimate was used for a given endpoint, its contributing underlying datasets were not simultaneously entered separately into the same meta-analysis. RCT arms are shown separately and were analyzed as comparative evidence rather than pooled with non-randomized proportions.

* In selected cohorts, multiple EGFR mutations were permitted and mutation categories were therefore non-mutually exclusive. Accordingly, an individual patient could contribute to more than one reported mutation category, and subtype percentages should not be summed to 100%. Mutation data are presented according to the definitions used in the original study reports.

After assessing population overlap, we identified five independent underlying non-randomized trial datasets across four reports considered for endpoint-specific quantitative synthesis (30–33): HERTHENA-Lung01, the updated U31402-A-U102 dataset, the Teliso-V Arm E dataset, and the two independent trial datasets contributing to the Ahn 2025 pooled Dato-DXd analysis (TROPION-Lung05 and TROPION-Lung01). When a pooled analytical estimate from the Ahn 2025 analysis was used for a given endpoint, its two contributing trial datasets were not entered separately into the same meta-analysis. Earlier or overlapping reports, including the Jänne 2022 U31402-A-U102 report and the original TROPION-Lung05 report, were retained for descriptive purposes but were not simultaneously included with their overlapping updated or pooled datasets. The two randomized controlled trials (34, 35), OptiTROP-Lung04 and OptiTROP-Lung03, were summarized separately as comparative evidence and were not pooled with non-randomized proportional outcomes.

The included reports spanned phase I/Ib through phase III clinical development and evaluated ADCs targeting TROP2, HER3, or c-MET. The investigated ADCs included datopotamab deruxtecan, patritumab deruxtecan, sacituzumab tirumotecan, and telisotuzumab vedotin. Across the reported cohorts and treatment arms, median age generally ranged from 55 to 65 years, and women accounted for approximately 52.2%–68.0% of participants. The proportion of patients with brain metastases ranged from 15.8% to 51.1%, reflecting differences in study eligibility criteria and enrolled populations. Prior osimertinib exposure varied from 44.5% to 100%, whereas prior platinum-based chemotherapy exposure ranged from 47.4% to 100% among cohorts with available data.

EGFR exon 19 deletion and L858R were the predominant mutation subtypes across most cohorts. Mutation subtype data were extracted according to the definitions used in the original study reports. In some cohorts, multiple EGFR mutations were permitted and the reported mutation categories were therefore non-mutually exclusive; consequently, the corresponding percentages should be interpreted descriptively and were not expected to sum to 100%.

### 3.3 Characteristics of ADCs included in clinical studies

Four ADCs were evaluated in the included studies: datopotamab deruxtecan, patritumab deruxtecan, sacituzumab tirumotecan, and telisotuzumab vedotin. These agents primarily targeted TROP2, HER3, and c-MET. Differences in antibody scaffold, linker design, and payload type may contribute to the observed variation in efficacy and safety profiles. The targets, biological characteristics, and primary payloads of each ADC are summarized in **Table 4**.

**Table 4:** Molecular design and pharmacological profiles of ADCs included in the systematic review.

ADC drug	Target	Payload	Linker	DAR	Bystander effect	Pharmacological profile/rationale in EGFR-TKI–resistant NSCLC
Datopotamab deruxtecan (Dato-DXd)	TROP2	DXd (topoisomerase I inhibitor)	GGFG tetrapeptide cleavable linker	~4	Strong	TROP2-mediated internalization followed by lysosomal cleavage and DXd release, inducing DNA damage and apoptosis; may be particularly relevant in resistant tumors with compensatory TROP2 expression and intratumoral heterogeneity.
Patritumab deruxtecan (HER3-DXd)	HER3 (ERBB3)	DXd (topoisomerase I inhibitor)	GGFG tetrapeptide cleavable linker	~8	Strong	HER3-mediated internalization with intracellular DXd release; mechanistically attractive in EGFR-TKI resistance because HER3 is commonly upregulated as a compensatory survival pathway and can sustain PI3K/AKT signaling.
Sacituzumab tirumotecan (Sac-TMT)	TROP2	KL610023 (belotecan derivative; topoisomerase I inhibitor)	Stabilized cleavable linker	~7.6	Strong	TROP2-directed delivery with dual pH/protease-sensitive release; the improved linker stability may reduce systemic exposure while preserving activity in TROP2-intermediate/low expression settings.
Telisotuzumab vedotin (Teliso-V)	c-MET	MMAE (microtubule inhibitor)	Val-Cit cleavable linker	~3.1	Moderate	c-MET-mediated internalization with MMAE release, leading to microtubule inhibition and G2/M arrest; biologically relevant in c-MET–driven bypass resistance or overexpression-mediated resistance.

ADC, antibody-drug conjugate; DAR, drug-to-antibody ratio; EGFR, epidermal growth factor receptor; NSCLC, non-small-cell lung cancer; TKI, tyrosine kinase inhibitor; TROP2, trophoblast cell-surface antigen 2; HER3, human epidermal growth factor receptor 3; c-MET, mesenchymal-epithelial transition factor.

The bystander effect of Teliso-V has been less consistently characterized across the literature and is therefore conservatively described as moderate.

### 3.4 Pooled efficacy and safety analysis of single-arm studies

#### 3.4.1 Objective response rate

Four non-overlapping analytical estimates from non-randomized evidence were included in the pooled ORR analysis. The pooled ORR was 39.2% (95% CI, 31.1%–48.0%), suggesting antitumor activity of ADC-based therapies in patients with previously treated EGFR-mutated NSCLC.

Substantial between-dataset heterogeneity was observed (I² = 70.5%), which may reflect differences in ADC targets, drug platforms, treatment settings, prior treatment exposure, and patient characteristics. Exploratory subgroup analysis according to ADC target suggested numerical variation across platforms; however, the between-subgroup difference was not statistically significant (p = 0.1427). Given the limited number of datasets within individual subgroups, particularly the single analytical estimate available for the TROP2- and c-MET-directed subgroups, these findings should be interpreted as exploratory and hypothesis-generating.

Overall, after exclusion of overlapping populations, the overlap-adjusted pooled analysis continued to support an antitumor signal for ADC-based strategies in this treatment setting, although interpretation should remain cautious given the substantial heterogeneity and limited number of available datasets (**Figure 3**).

**Figure 3:**
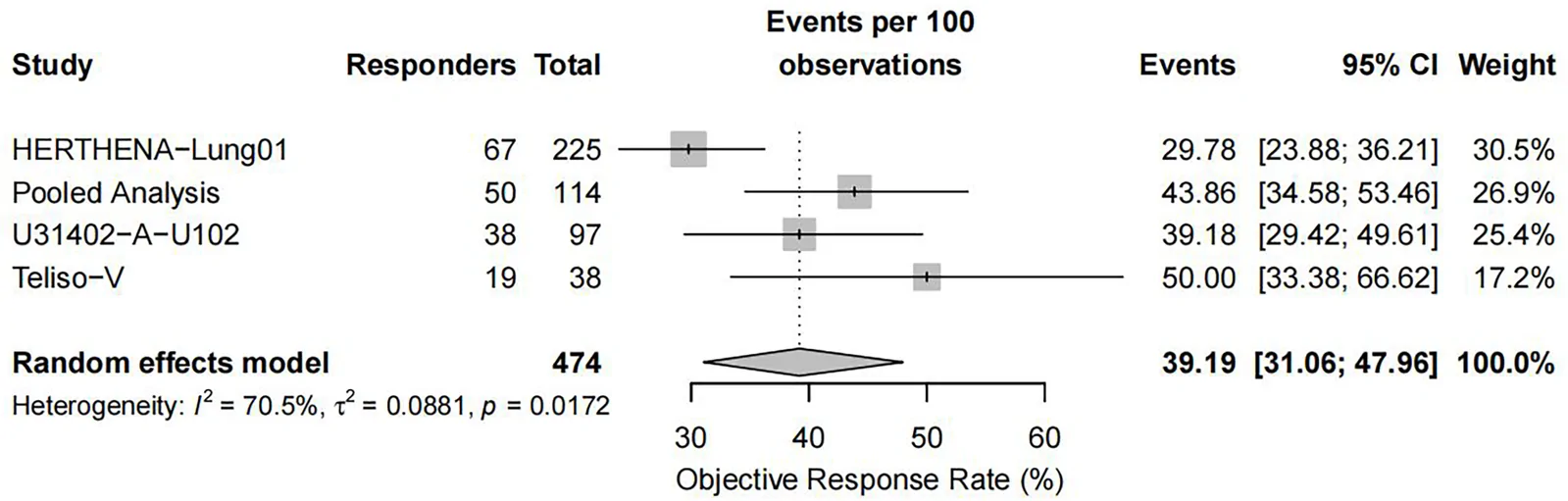
Pooled objective response rate (ORR) of ADCs in EGFR-mutated NSCLC after EGFR-TKI resistance. Individual studies are represented by squares, with square size proportional to study weight. Horizontal lines indicate 95% confidence intervals (CIs). Diamonds indicate pooled estimates from the random-effects model.

#### 3.4.2 Disease control rate

Four non-overlapping analytical estimates from non-randomized evidence were included in the pooled DCR analysis.The pooled DCR was 78.9% (95% CI, 72.3%–84.2%).

The DCR analysis showed lower between-dataset heterogeneity than the ORR analysis (I² = 54.3%), suggesting greater consistency in disease-control estimates.

Exploratory subgroup analysis according to ADC target showed comparable disease control activity among different ADC categories. However, because some subgroups were represented by only one dataset, these findings were considered exploratory (**Figure 4**)

**Figure 4:**
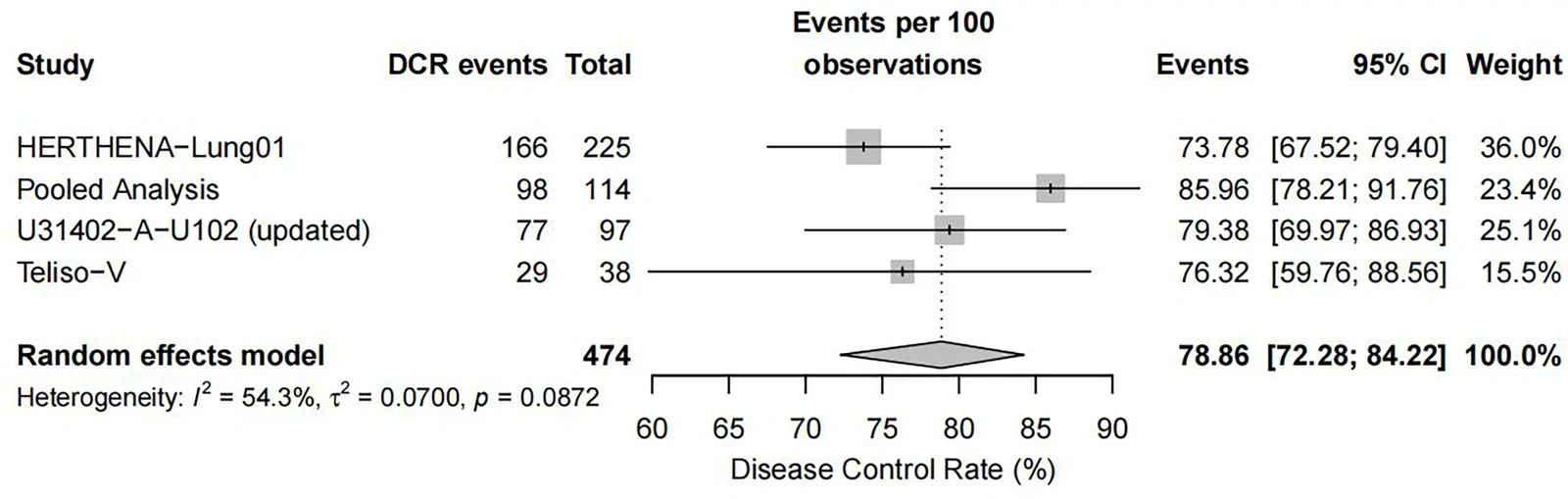
Pooled disease control rate (DCR) of ADCs in EGFR-mutated NSCLC after EGFR-TKI resistance. Individual studies are represented by squares, with square size proportional to study weight. Horizontal lines indicate 95% CIs. Diamonds indicate pooled estimates from the random-effects model.

#### 3.4.3 Grade ≥3 treatment-emergent adverse events

Three non-overlapping analytical datasets reporting grade ≥3 TEAEs were included in the pooled safety analysis (**Figure 5**). The pooled incidence of grade ≥3 TEAEs was 65.2% (95% CI, 50.2%–77.7%) using a random-effects model.

**Figure 5:**
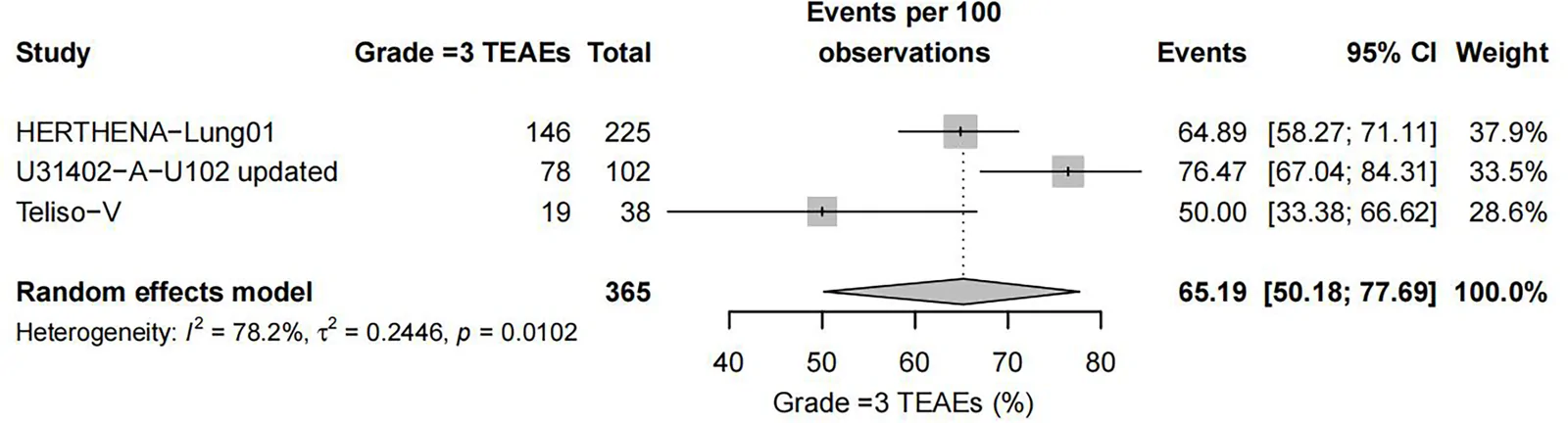
Pooled incidence of grade ≥3 treatment-emergent adverse events (TEAEs) in non-randomized ADC datasets. Individual datasets are represented by squares, with square size proportional to study weight. Horizontal lines indicate 95% confidence intervals (CIs), and the diamond represents the pooled estimate from the random-effects model. (6).

Substantial heterogeneity was observed among datasets (I² = 78.2%, P = 0.010), which may reflect differences in ADC platforms, treatment regimens, safety assessment criteria, and patient characteristics.

Leave-one-out sensitivity analysis demonstrated that the pooled estimate remained generally consistent after sequential exclusion of individual datasets, suggesting that no single dataset had a disproportionate influence on the overall result.

#### 3.4.4 Interstitial lung disease

Four non-overlapping analytical datasets were included in the ILD analysis. The pooled reported incidence of ILD was 6.0% (95% CI, 4.1%–8.5%), with no detected statistical heterogeneity (I² = 0%). Sensitivity analysis excluding the Teliso-V dataset yielded a similar pooled incidence of 5.8% (95% CI, 3.9%–8.4%; I² = 0%), indicating numerical stability of the overall estimate. Nevertheless, given the limited number of datasets and differences in event ascertainment across studies, the absence of statistical heterogeneity should be interpreted cautiously (**Figure 6**).

**Figure 6:**
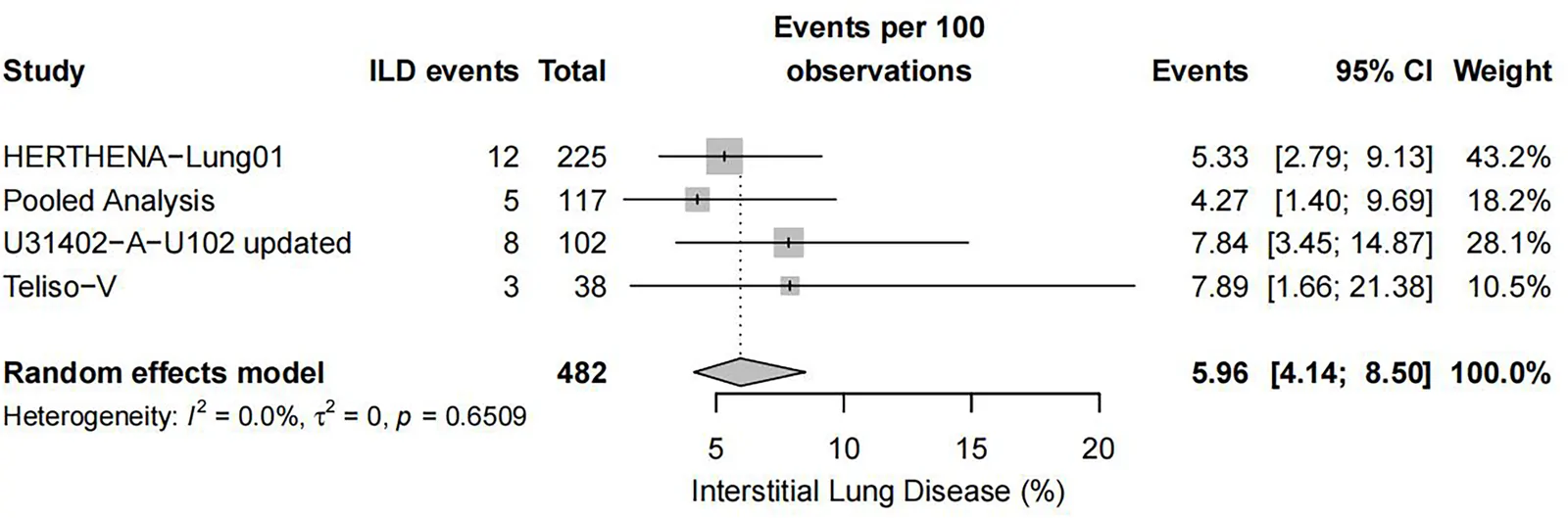
Incidence of interstitial lung disease (ILD) with ADCs in EGFR-mutated NSCLC after EGFR-TKI resistance. Individual studies are represented by squares, with square size proportional to study weight. Horizontal lines indicate 95% CIs. Diamonds indicate pooled estimates. Both common-effect and random-effects models are presented because between-study heterogeneity was negligible.

### 3.5 Comparative evidence from randomized controlled trials

#### 3.5.1 OptiTROP-Lung04

OptiTROP-Lung04 was a phase III randomized controlled trial that enrolled patients with EGFR-mutated advanced nonsquamous NSCLC whose disease had progressed after EGFR-TKI therapy. In the sacituzumab tirumotecan (sac-TMT) group, BIRC-assessed ORR was 60.6%, DCR was 87.2%, and median PFS was 8.3 months, all of which were superior to those observed with pemetrexed plus platinum chemotherapy (ORR, 43.1%; DCR, 80.3%; median PFS, 4.3 months). The corresponding PFS HR was 0.49 (95% CI, 0.39-0.62), and the OS HR was 0.60 (95% CI, 0.44-0.82; P = 0.001), indicating that sac-TMT significantly reduced the risk of disease progression or death and showed a survival benefit. In terms of safety, grade ≥3 TRAEs occurred in 58.0% of patients in the sac-TMT group, and serious TRAEs occurred in 9.0%, suggesting that the overall safety profile was manageable.

#### 3.5.2 OptiTROP-Lung03

OptiTROP-Lung03 was a phase II randomized controlled trial that enrolled patients with EGFR-mutated NSCLC whose disease had progressed after EGFR-TKI and platinum-based chemotherapy. In the sac-TMT group, BIRC-assessed ORR was 45%, DCR was 82%, and median PFS was 6.9 months, all of which were superior to those observed with docetaxel (ORR, 16%; DCR, 60%; median PFS, 2.8 months). The HR was 0.30 for PFS (95% CI, 0.20-0.46; P < 0.001) and 0.49 for OS (95% CI, 0.27-0.88; P = 0.007), indicating a clear relative efficacy advantage for sac-TMT in this population. The incidence of grade ≥3 TRAEs was lower with sac-TMT than with docetaxel (56% vs. 72%), suggesting that improved efficacy was not achieved at the expense of unacceptable high-grade toxicity.

#### 3.5.3 Integrated interpretation of randomized controlled evidence

Taken together, the two RCTs provided comparative evidence for sac-TMT across distinct treatment settings. However, because the available randomized evidence was insufficient to support a robust network meta-analysis or cross-trial quantitative pooling, the two RCTs were presented separately and synthesized narratively using trial-specific comparative estimates (**Table 5**)

**Table 5:** Comparative efficacy and safety evidence from randomized controlled trials of sac-TMT.

Study	Cohort/arm	Phase	Population	Randomization	Sample size (ITT)	Analysis set	Primary endpoint	ORR (BIRC)	DCR (BIRC)	mPFS(BIRC)	PFS HR (BIRC)	mOS	OS HR	≥3 grade TRAEs	Serious TRAEs	Comparator
OptiTROP-Lung04	sac-TMT Group	Phase III	Advanced non-squamous NSCLC with EGFR mutations (19del/L858R), progressed after EGFR-TKI (1st/2nd/3rd generation allowed)	1:1	188	ITT	PFS assessed by BIRC	60.6% (114/188) [95%CI 53.3-67.7%]	87.2% (164/188) [95%CI 81.6-91.6%]	8.3 months (95%CI 6.7-9.9)	0.49 (95%CI 0.39-0.62)	NR (18-month OS rate 65.8% [95%CI 58.3-72.3%])	0.60 (95%CI 0.44-0.82), p=0.001	58.0% (109/188)	9.0% (17/188)	Pemetrexed + platinum
	Chemotherapy Group (pemetrexed + platinum)	Phase III	same	1:1	188	ITT	–	43.1% (81/188) [95%CI 35.9-50.5%]	80.3% (151/188) [95%CI 73.9-85.7%]	4.3 months (95%CI 4.2-5.5)	–	17.4 months (18-month OS rate 48.0% [95%CI 40.2-55.4%])	–	53.8% (98/182)	17.6% (32/182)	–
OptiTROP-Lung03	sac-TMT Group	Phase II	Advanced NSCLC with EGFR mutations (19del/L858R), progressed after EGFR-TKI + platinum-based chemotherapy	2:1	91	ITT (primary analysis); safety set (all who received ≥1 dose)	ORR assessed by BIRC	45% (41/91) [95%CI 35-56%]	82% (75/91) [95%CI 73-90%]	6.9 months (95%CI 5.4-8.2)	0.30 (95%CI 0.20-0.46)	NR (12-month OS rate 73% [95%CI 62-81%])	0.49 (95%CI 0.27-0.88)	56% (51/91)	16% (15/91)	Docetaxel
	Docetaxel Group	Phase II	same	2:1	46	same as above	–	16% (7/45) [95%CI 7-30%]	60% (27/45) [95%CI 44-74%]	2.8 months (95%CI 1.6-4.1)	–	NR (12-month OS rate 54% [95%CI 39-67%])	–	72% (33/46)	41% (19/46)	–

Values are presented as n (%) unless otherwise indicated. BIRC, blinded independent central review; EGFR, epidermal growth factor receptor; ITT, intention-to-treat; mOS, median overall survival; mPFS, median progression-free survival; NR, not reached; ORR, objective response rate; OS, overall survival; Q3W, every 3 weeks; RCT, randomized controlled trial; sac-TMT, sacituzumab tirumotecan; TKI, tyrosine kinase inhibitor; TRAEs, treatment-related adverse events.

For OptiTROP-Lung04, crossover was not allowed. For OptiTROP-Lung03, crossover from docetaxel to sac-TMT after progression was permitted, if applicable.

## 4 Discussion

### 4.1 Main findings and clinical implications

This study systematically synthesized the available clinical evidence on ADCs for EGFR-mutated NSCLC after EGFR-TKI resistance. Overall, ADCs showed clear antitumor activity in this population. Pooled analyses of non-randomized datasets yielded ORR and DCR estimates of 39.2% and 78.9% (30–33), respectively.Beyond these response-based outcomes, time-to-event outcomes from the non-randomized single-arm studies were reported as study-specific Kaplan–Meier estimates, with median PFS ranging from 5.5 to 7.4 months and reported median OS ranging from 11.9 to 15.8 months. Because these studies lacked internal comparators, comparative HRs could not be estimated, and these survival outcomes were therefore interpreted descriptively rather than quantitatively pooled. By contrast,two randomized controlled trials showed that sac-TMT improved ORR and PFS compared with standard therapy and also suggested an OS benefit (34, 35). These findings indicate that the clinical value of ADCs is increasingly supported by emerging randomized evidence. However, given that current comparative data remain limited to a small number of randomized trials, further validation from additional studies and longer follow-up is required before definitive treatment positioning can be established.

Importantly, efficacy and safety differed across ADC platforms. Observed heterogeneity in ORR, DCR, and high-grade safety outcomes across datasets and ADC platforms indicates that ADCs should not be treated as a uniform therapeutic class (19). Therefore, the pooled estimates presented in this study should be interpreted as an overall assessment of ADC-based therapeutic strategies in EGFR-TKI-resistant NSCLC rather than as evidence of the superiority of one ADC platform over another.Their clinical performance may be shaped by target biology, payload structure, linker design, and dosing schedule (36). Therefore, later-line ADC selection should focus on identifying which ADC is most appropriate for which patient subgroup (37).

Ongoing clinical development and regulatory progress in NSCLC further support this view. In 2025, regulatory progress in NSCLC was supported by clinical evidence for datopotamab deruxtecan in previously treated EGFR-mutated NSCLC and for telisotuzumab vedotin in c-MET protein–overexpressing nonsquamous NSCLC (18, 38, 39).Notably, telisotuzumab vedotin is used in relation to c-MET protein expression, indicating that ADCs are evolving from broadly applied therapeutic candidates toward biomarker-informed treatment platforms (38). Accordingly, the major question in ADC development is shifting from whether these agents are active to how patients should be selected and how treatment should be sequenced.

In this review, sac-TMT showed consistent benefit in randomized controlled trials,particularly for ORR and PFS (34, 35). Unlike single-arm studies, which primarily provide absolute estimates of efficacy, randomized evidence demonstrated that ADC activity can translate into relative benefit over standard therapy. However, OS requires more cautious interpretation because it may be influenced by post-progression treatment and crossover. This issue is particularly relevant to OptiTROP-Lung03, in which patients assigned to docetaxel were permitted to cross over to sac-TMT after confirmed disease progression. As 36.4% of control-arm patients subsequently received sac-TMT, the ITT OS contrast may have been attenuated by post-progression exposure to active study treatment. Consistent with this possibility, the reported ITT OS HR was 0.49, whereas the prespecified RPSFT crossover-adjusted analysis yielded an HR of 0.36 (34). However, because crossover-adjusted estimates depend on modeling assumptions, the ITT HR remains the primary randomized estimate, while the adjusted analysis should be regarded as supportive evidence that crossover may have diluted the observed OS treatment contrast. Accordingly, the OS findings support a potential survival benefit but should not be interpreted as a definitive estimate of its magnitude. Overall, the randomized trials provide higher-level evidence supporting the potential role of sac-TMT in EGFR-TKI-resistant NSCLC, while additional randomized evidence and longer follow-up remain necessary to further define its survival benefit and treatment positioning.

### 4.2 Biological interpretation of inter-platform heterogeneity

The observed heterogeneity in efficacy and toxicity across ADC platforms suggests that the clinical performance of ADCs is influenced by multiple biological factors. After EGFR-TKI failure, tumor-cell membrane proteins such as HER3 (21), TROP2 (40), and c-MET (41) may remain expressed and can function as targets for ADC-mediated delivery of cytotoxic payloads. This mechanism may allow ADCs to retain antitumor activity in the setting of EGFR-TKI resistance.

Differences in antibody structure, linker stability, and payload type may also contribute to inter-platform variability (42). For example, the bystander effect of DXd-based ADCs may enhance coverage of intratumoral heterogeneity and reduce escape by antigen-low clones (43). By contrast, c-MET-targeted ADCs may be most relevant in tumors with c-MET overexpression or bypass activation. Thus, the therapeutic value of ADCs likely depends not only on target expression but also on platform-specific drug-delivery properties and the underlying resistance context.

The relationship between target expression and efficacy is unlikely to be strictly linear. In a pooled study, sac-TMT demonstrated antitumor activity in both study-defined TROP2-high and TROP2-low subgroups. Among 92 biomarker-evaluable patients, the ORR was 39% in the TROP2-high subgroup and 31% in the TROP2-low subgroup, while the median PFS was 10.9 months and 7.2 months, respectively (log-rank p = 0.020) (44).These findings suggest that lower TROP2 expression did not preclude response, whereas higher TROP2 expression may be associated with longer PFS. However, because this was an exploratory analysis of non-randomized studies that included patients with and without EGFR mutations, these findings do not establish TROP2 expression as a definitive predictive biomarker for sac-TMT in EGFR-mutated NSCLC (44).Future precision use of ADCs may therefore require integrated assessment of target expression, membrane localization, tumor heterogeneity, payload properties, and prior treatment history rather than reliance on a single expression threshold.

### 4.3 Safety considerations and treatment selection

The pooled incidence of grade ≥3 TEAEs was 65.2%, with substantial between-dataset heterogeneity (I² = 78.2%), indicating variation in high-grade toxicity across the included ADC datasets. This heterogeneity may reflect differences in ADC platforms, treatment regimens, safety-assessment criteria, and patient characteristics (42, 45).Accordingly, the pooled estimate should be interpreted as a class-level summary rather than as the expected toxicity profile of any individual ADC.

ILD requires particular attention during ADC therapy. In this study, the pooled reported incidence of ILD was 6.0% (95% CI, 4.1%–8.5%), with no detected statistical heterogeneity (I² = 0%). Sensitivity analysis excluding the Teliso-V dataset yielded a similar estimate of 5.8% (95% CI, 3.9%–8.4%; I² = 0%), supporting the numerical stability of the pooled result. However, the relatively low and statistically consistent reported incidence should not be interpreted as definitive evidence of uniformly low underlying risk. ILD ascertainment may have varied across studies because of differences in event definitions, treatment attribution, adjudication procedures, grading practices, surveillance intensity, and follow-up duration. Such variability may lead to underrecognition of low-grade or subclinical events. Moreover, because only a small number of analytical datasets contributed to the pooled analysis, the absence of statistical heterogeneity does not establish complete clinical or methodological homogeneity. Accordingly, the pooled estimate is best interpreted as a summary of reported ILD incidence across heterogeneous trial-specific ascertainment frameworks rather than as a precise estimate of the underlying biological risk across all ADC platforms. Given the potential for treatment discontinuation and fatal outcomes, careful symptom assessment, close clinical monitoring, and appropriate imaging surveillance remain essentia (46).

In the randomized controlled trials included in this review, improved efficacy with sac-TMT was not accompanied by a higher incidence of grade ≥3 TRAEs than control therapy. These findings suggest that ADC benefit does not necessarily require a substantial increase in high-grade toxicity and support the feasibility of ADC use in subsequent-line treatment.

These platform-specific toxicity patterns may inform patient selection, although they should not be treated as definitive prescribing rules because no head-to-head trial has directly compared the available ADCs. HER3-DXd is characterized predominantly by hematologic toxicity, particularly thrombocytopenia and neutropenia, together with a recognized risk of ILD (30); patients with preserved bone marrow reserve and without active or previously clinically significant ILD may be better positioned to tolerate this toxicity profile, whereas baseline cytopenias or limited pulmonary reserve warrant caution. Dato-DXd is particularly associated with oral mucositis/stomatitis, ocular surface events, and ILD (31); patients without clinically significant oral mucosal or corneal/ocular surface disease who can adhere to preventive oral care and ophthalmologic monitoring may be better candidates (47).The main treatment-related toxicities reported with sac-TMT were hematologic, including neutropenia or leukopenia and anemia, while stomatitis was also observed (33, 34). In OptiTROP-Lung03, grade ≥3 TRAEs occurred less frequently with sac-TMT than with docetaxel (56% vs. 72%) (34).Teliso-V is primarily relevant to c-MET protein-overexpressing tumors, but peripheral neuropathy and edema are frequent; clinically significant baseline neuropathy, troublesome edema, or hypoalbuminemia would warrant particular caution when considering this platform (33).

Overall, ADC selection should integrate target eligibility and evidence of antitumor benefit with the individual patient’s bone marrow reserve, pulmonary status, oral and ocular health, neurologic function, prior treatment exposure, and comorbidities. These toxicity-informed considerations are intended to support individualized treatment rather than establish a definitive hierarchy among ADC platforms and require validation in prospective comparative studies.

### 4.4 Limitations and future directions

Several limitations should be acknowledged. First, most available evidence was derived from non-randomized datasets, which can characterize absolute efficacy and toxicity but cannot support causal inference because of the absence of concurrent control group (48, 49).

Second, potential overlap among clinical reports represented an additional methodological consideration. Some publications originated from updated analysis, subgroup analysis, or pooled datasets derived from related clinical trial populations. Although these reports provide more mature and comprehensive information, simultaneous inclusion of overlapping datasets may lead to double counting of patients. In this study, relationships among reports were carefully assessed before quantitative synthesis, and endpoint-specific datasets were selected to preserve patient independence and avoid duplication.

Third, pooling distinct ADC platforms—each with unique biological mechanisms, payloads (e.g., topoisomerase I inhibitors vs. microtubule inhibitors), and specific toxicity profiles (e.g., differing risks of ILD or myelosuppression)—represents an inherent limitation of this meta-analysis. Consequently, the pooled estimates generated from the non-randomized datasets should be interpreted as a broad, class-level baseline for ADCs rather than an expected absolute outcome for any single agent. Variations in target type, prior treatment line, brain metastasis proportion, and molecular background across studies may have further contributed to inter-study heterogeneity and affected the stability of the pooled results. Furthermore, although two RCTs provided higher-level comparative evidence, the randomized evidence base remained limited to two clinically heterogeneous trials of the same ADC. This precluded a robust network meta-analysis and limited indirect comparisons across ADC platforms.

Fourth, formal assessment of publication bias was not feasible because most pooled analyses included fewer than five datasets. Although comprehensive literature searches, predefined eligibility criteria, risk-of-bias assessment, and sensitivity analysis were performed to minimize potential bias, the possibility of residual publication bias cannot be completely excluded.

Future research should move beyond confirming ADC activity and focus on precision stratification and optimal treatment sequencing. Key priorities include identifying predictive biomarkers for patient selection, defining optimal sequencing strategies among ADCs, EGFR-TKIs, chemotherapy, and immunotherapy, and integrating ctDNA, brain metastasis status, and patient-reported outcomes into more comprehensive predictive models (50). Real-world evidence, long-term safety monitoring, and cost-effectiveness analysis will also be important for clinical implementation.

## 5 Conclusion

ADCs demonstrate antitumor activity in previously treated EGFR-mutated NSCLC after EGFR-TKI resistance, while emerging randomized evidence provides comparative support for sac-TMT, particularly for ORR and PFS. However, efficacy and toxicity vary across ADC platforms, and the pooled estimates should be interpreted as class-level summaries rather than as direct comparisons between individual agents. Additional randomized trials, biomarker-informed patient selection, and longer follow-up are needed to define the optimal positioning, long-term safety, and survival benefit of individual ADCs.

## Data Availability

The original contributions presented in the study are included in the article/**Supplementary Material**. Further inquiries can be directed to the corresponding author.
